# Predicting hepatocellular carcinoma outcomes and immune therapy response with ATP-dependent chromatin remodeling-related genes, highlighting MORF4L1 as a promising target

**DOI:** 10.1186/s12935-024-03629-2

**Published:** 2025-01-05

**Authors:** Chao Xu, Litao Liang, Guoqing Liu, Yanzhi Feng, Bin Xu, Deming Zhu, Wenbo Jia, Jinyi Wang, Wenhu Zhao, Xiangyu Ling, Yongping Zhou, Wenzhou Ding, Lianbao Kong

**Affiliations:** 1 Hepatobiliary Center, The First Affiliated Hospital of Nanjing Medical University, Key Laboratory of Liver Transplantation, Chinese Academy of Medical Sciences, NHC Key Laboratory of Hepatobiliary Cancers, Nanjing, Jiangsu China; 2 Children’s Hospital of Nanjing Medical University, No. 72, Guangzhou Road, Nanjing, 210008 Jiangsu China; 3 Department of Hepatobiliary and Pancreatic Surgery, Affiliated Hospital of Qingdao University, Qingdao, China; 4 Department of Hepatobiliary Surgery, Wuxi No.2 People’s Hospital, No. 68 Zhongshan Road, Wuxi, China

**Keywords:** ATP-dependent chromatin remodeling, Hepatocellular carcinoma, Cancer stem cells, Immunotherapy

## Abstract

**Background:**

Hepatocellular carcinoma (HCC) continues to be a major cause of cancer-related death worldwide, primarily due to delays in diagnosis and resistance to existing treatments. Recent research has identified ATP-dependent chromatin remodeling-related genes (ACRRGs) as promising targets for therapeutic intervention across various types of cancer. This development offers potential new avenues for addressing the challenges in HCC management.

**Methods:**

This study integrated bioinformatics analyses and experimental approaches to explore the role of ACRRGs in HCC. We utilized data from The Cancer Genome Atlas (TCGA) and the Gene Expression Omnibus (GEO), applying machine learning algorithms to develop a prognostic model based on ACRRGs’ expression. Experimental validation was conducted using quantitative real-time Polymerase Chain Reaction (qRT-PCR), Western blotting, and functional assays in HCC cell lines and xenograft models.

**Results:**

Our bioinformatics analysis identified four key ACRRGs—MORF4L1, HDAC1, VPS72, and RUVBL2—that serve as prognostic markers for HCC. The developed risk prediction model effectively distinguished between high-risk and low-risk patients, showing significant differences in survival outcomes and predicting responses to immunotherapy in HCC patients. Experimentally, MORF4L1 was demonstrated to enhance cancer stemness by activating the Hedgehog signaling pathway, as supported by both in vitro and in vivo assays.

**Conclusion:**

ACRRGs, particularly MORF4L1, play crucial roles in modulating HCC progression, offering new insights into the molecular mechanisms driving HCC and potential therapeutic targets. Our findings advocate for the inclusion of chromatin remodeling dynamics in the strategic development of precision therapies for HCC.

**Supplementary Information:**

The online version contains supplementary material available at 10.1186/s12935-024-03629-2.

## Introduction

HCC is recognized as the sixth most common cancer globally and the third leading cause of cancer-related deaths. Over recent decades, an upward trajectory in HCC incidence has been documented [1]. Projections for the United States indicate an anticipated diagnosis of 41,630 new HCC cases by 2024, with an estimated 29,840 fatalities attributable to the disease within the same timeframe [2]. While surgical resection and ablation represent cornerstone treatments for potentially curing early-stage HCC, recent advancements in targeted therapies and immunotherapies have introduced new avenues for patients with inoperable conditions. However, the effectiveness of these treatments is curtailed by challenges in early HCC detection and complications stemming from drug resistance and recurrence [3].

ATP-dependent chromatin remodeling is pivotal in regulating gene expression and is critically involved in cancer development. This remodeling process utilizes various complexes, such as SWI/SNF, which employ ATP hydrolysis to modify chromatin structures, thereby influencing transcriptional activities. Depending on the specific genetic alterations and cellular context, this remodeling can either contribute to or inhibit tumorigenesis. Significant research emphasizes the essential nature of changes in chromatin remodeling components in both the onset and progression of cancer [4, 5].

Targeting the ATPases within these remodeling complexes presents a promising therapeutic avenue, particularly in cancers with a high dependency on enhancer activity. In this context, the regulation of transcriptional enhancers is crucial [6]. Additionally, recent studies have highlighted the extensive impact of ATP-dependent chromatin remodeling on tumor behavior and resistance to therapies [7, 8].

The complexity and critical importance of ATP-dependent chromatin remodeling in the pathology of cancer underscore its value as a target for precision medicine approaches. These strategies aim to disrupt the aberrant chromatin states that are fundamental to cancer progression, offering new possibilities for effective cancer therapy.

This investigation probes the involvement of ACRRGs in HCC. Our findings reveal a predominant overexpression of ACRRGs in HCC tissues, with implications for predicting patient overall survival. Leveraging machine learning and bioinformatics approaches, we have devised a prognostic model predicated on ACRRGs’ expression, validated externally for its predictive accuracy regarding patient survival outcomes. Additionally, through enrichment analysis and immune response analyses, our study suggests a contributory role of ATP-dependent chromatin remodeling in fostering HCC stemness and facilitating immune evasion. Further integration of single-cell data and experimental validation identified the MORF4L1 gene as a promoter of HCC stemness through activation of the Hedgehog signaling pathway. A flow chart of this research is shown in Figure S1.

## Methods and materials

### Next-generation sequencing data and single-cell data

The HCC cohort (TCGA-LIHC) data, encompassing sequencing, mutation, and clinical information, were sourced from the TCGA. Additionally, hcc cohort data (GSE14520) and single-cell dataset (GSE149614), along with their respective clinical information, were retrieved from the GEO. A compilation of 113 ACRRGs was derived from the Kyoto Encyclopedia of Genes and Genomes (KEGG) and is presented in Table S1. Furthermore, the reference expression matrix for 22 types of immune cells was adopted from a prior study [9].

### Machine learning and bioinformatics algorithm for risk prediction in HCC

We employed the DESeq2 algorithm to identify differentially expressed genes (DEGs) between tumor tissues and adjacent non-tumoral tissues within the TCGA-LIHC cohort. An intersection of these DEGs with ACRRGs was performed to identify differentially expressed genes related to ATP-dependent chromosomal remodeling (ACRRDEGs). Subsequent analysis utilized the Lasso regression algorithm to select pivotal ACRRDEGs, which were foundational in developing a HCC risk prediction model. This model was constructed using multivariate Cox regression analysis, assigning each patient a risk score. Patients were categorized into high and low-risk groups based on the median score, facilitating a nuanced evaluation of their prognostic outcomes. The model's robustness and predictive validity were assessed through Receiver Operating Characteristic (ROC) curve analysis. Further validation was conducted using an additional HCC cohort (GSE14520), reinforcing the model's applicability. Moreover, the prognostic relevance of the risk score, alongside other clinical parameters in HCC, was meticulously examined through multivariate Cox regression analysis. This led to the creation of nomograms that integrate these prognostically significant indicators, offering a comprehensive tool to predict patient outcomes. The predictive accuracy of these nomograms, along with the individual indicators, was quantitatively evaluated using ROC curve analysis and the time-dependent concordance index.

### Analysis of functional enrichment and immune responses

To discriminate between high and low-risk groups of hepatocellular carcinoma patients, we implemented DESeq2 for the identification of DEGs. Subsequently, these DEGs were analyzed through Gene Set Enrichment Analysis (GSEA) to delineate critical biological pathways, employing a methodology analogous to that used in investigating the downstream pathways associated with MORF4L1. The composition of immune cells and immune scores within these patient cohorts were evaluated using CIBERSORT and ESTIMATE, respectively. Furthermore, the TIDE web resource was employed for forecasting the potential responsiveness of patients in both risk groups to immunotherapeutic interventions.

### Single-cell sequencing analysis and pseudo-temporal analysis

We processed single-cell sequencing data  employing the Seurat standard workflow, which includes quality control, normalization, dimensionality reduction, cell clustering, and cell identification. Subsequently, Cytotrace was used for pseudo-temporal analysis on normal liver cells, malignant cells, and cancer stem cells. This analysis aimed to pinpoint crucial genes driving the progression of HCC stemness.

### Hepatocellular carcinoma samples and cell lines

Our study utilized 65 matched pairs of cancerous and adjacent non-cancerous tissue samples from the First Affiliated Hospital of Nanjing Medical University. Ethical approval and written consent were obtained from all participants. All cell lines were acquired from the Shanghai Institute of Cell Biology, Chinese Academy of Sciences, and were cultured in Dulbecco's Modified Eagle Medium (DMEM) supplemented with 10% fetal bovine serum (FBS), at 37 °C in a 5% CO2 atmosphere.

### QRT-PCR

RNA was isolated using TRIzol reagent (Invitrogen), and its concentration was determined spectrophotometrically. Complementary DNA synthesis was conducted using a reverse transcription kit (VAzyme, Nanjing, China), followed by quantitative PCR analysis with Ace qPCR SYBR Green Master Mix (VAzyme) on an ABI 7900 PCR system (Applied Biosystems Inc., USA). GAPDH was used as the internal control, and primer sequences are listed in Supplementary Table S2.

### Lentiviral transfection

Lentiviruses for MORF4L1 overexpression, MORF4L1 knockdown (shMORF4L1), and controls were sourced from GenePharma (Shanghai, China). Transfections followed the manufacturer's guidelines, with puromycin selection at 2 μg/ml to establish stable cell lines.

### Western blot

Proteins were extracted, separated by SDS-PAGE, and transferred to PVDF membranes. After blocking with 5% non-fat milk, membranes were incubated with primary antibodies overnight at 4°C, followed by appropriate secondary antibodies. Detection used an enhanced chemiluminescence (ECL) system, with primary antibodies detailed in Supplementary Material, Table S3.

### Functional assays

We conducted a series of assays to evaluate cell phenotypes, including colony formation, EdU, cell viability, transwell migration and invasion, sphere formation, and limiting dilution assays. Each assay followed standardized protocols appropriate for the assessment of HCC cell behavior and stemness characteristics. The details of these assays are available in Supplementary File 1.

### Xenograft nude mouse model

Animal experiments adhered to protocols approved by the Institutional Animal Care and Use Committee at the First Affiliated Hospital of Nanjing Medical University. Five-week-old male BALB/c nude mice were divided into four groups, with six mice per group. Each mouse was inoculated with 1 million lentivirus-transfected cells in the axilla of the right upper limb. Subcutaneous tumor volumes were monitored over 30 days, after which the tumors were excised post-euthanasia. The removed tumors were weighed and analyzed immunohistochemicallly. An additional cohort of mice was randomly divided into four groups of four mice each, inoculated with 10 million lentivirus-stably transfected cells. By day 15, the average tumor volume had reached approximately 0.35 cm^3^. Subsequently, the mice were treated daily with oral Lenvatinib (20 mg/kg) for one week. Changes in subcutaneous tumor volume were monitored until day 45 when the mice were euthanized.

### Lung metastasis model

In this model, five-week-old male BALB/c nude mice were assigned randomly into four groups, with six mice per group. Each mouse received a tail vein injection of 1.2 million virally stable-transfected HCC cells. After 25 days, the mice were euthanized, and their lungs were harvested for photographic documentation, counting metastatic foci and hematoxylin and eosin (H&E) staining.

### Statistical analysis

Data were analyzed using appropriate statistical tests, with significance set at P < 0.05. Bioinformatics analyses utilized R software, while experimental data analyses employed Prism 8.0.2.

## Results

### ACRRGs show elevated expression in hcc indicating potential as prognostic markers for HCC

**Fig. 1:**
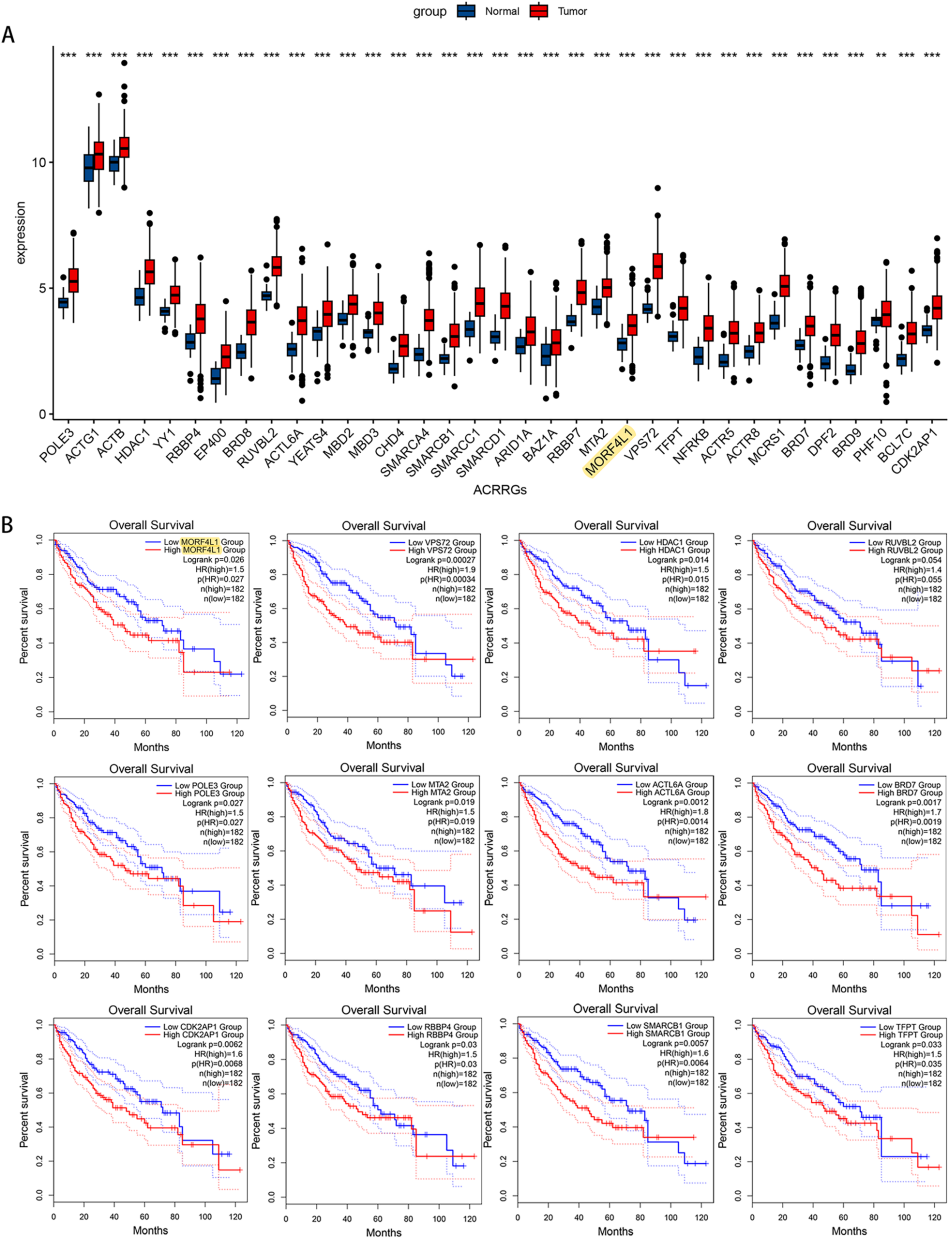
ATP-dependent chromatin remodeling-related genes (ACRRGs) exhibit elevated expression levels in tumor tissues compared to normal tissues, serving as potential prognostic markers for HCC. **A** Expression levels of ACRRGs in cancerous and adjacent non-tumor tissues in the TCGA database. **B** The impact of high versus low expression of ACRRGs on overall survival in HCC

We extracted data on genes involved in the ATP-dependent chromatin remodeling pathway from the KEGG database. By comparing their expression in HCC tissues to adjacent non-tumorous liver samples, and evaluating their prognostic significance through the TCGA database, we observed that the majority of ACRRGs exhibited elevated mRNA levels in HCC samples (Fig. 1A and Figure S2). Univariate Cox regression analysis indicated that a significant subset of these genes could serve as predictors for HCC patient survival (Fig. 1B and Figure S3).

### Construction and validation of a prognostic model for hepatocellular carcinoma based on ACRRDEGs

**Fig. 2:**
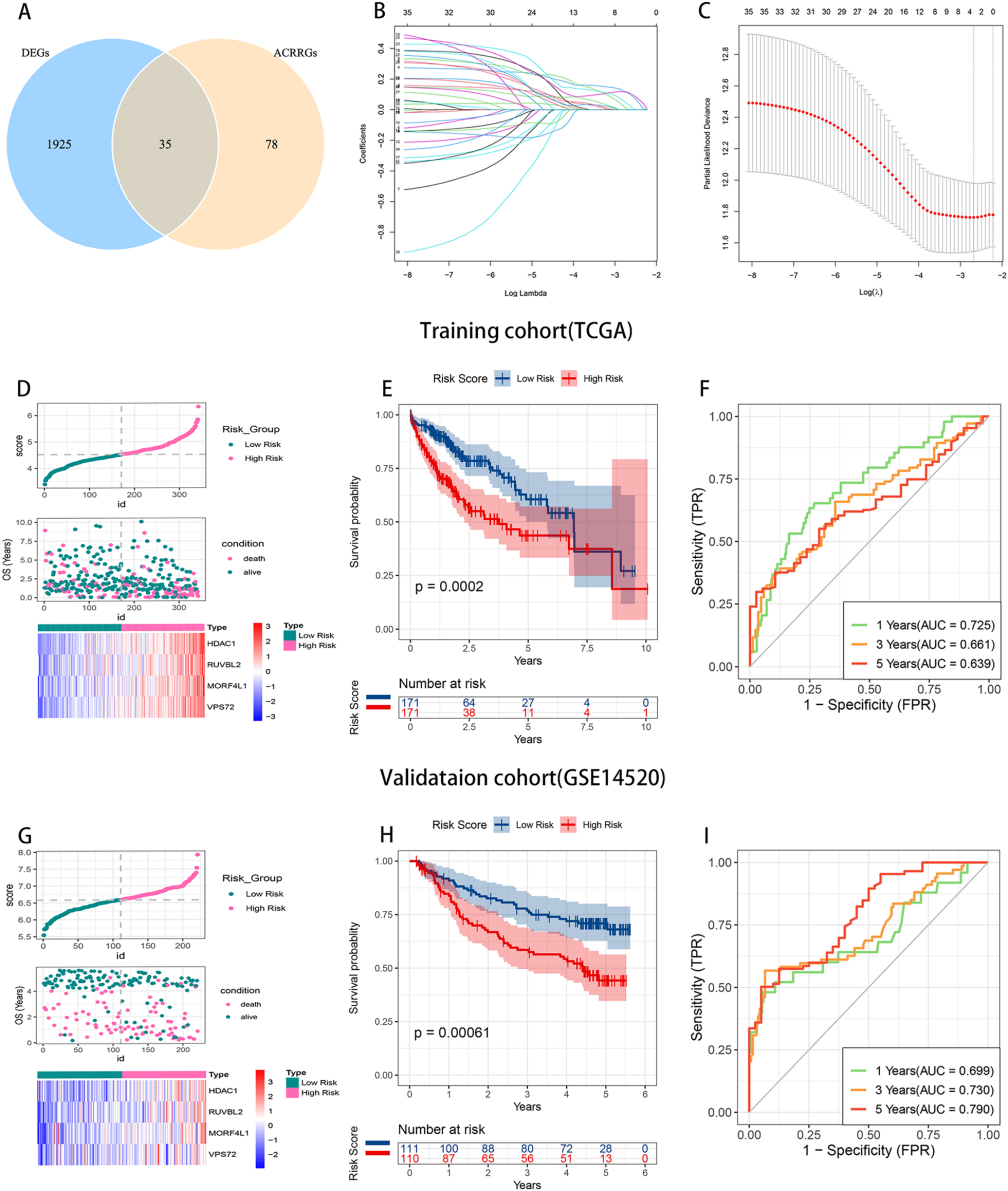
The risk model based on ACRRDEGs effectively forecasts the overall survival of HCC patients. **A** Intersection of differentially expressed genes (DEGs) from tumor and adjacent normal tissues with ACRRGs. **B** and **C** The process of selecting key genes using the LASSO method. **D** and **G** HCC patients are scored using the model, with scores determining assignment to either a high-risk or low-risk group. Survival proportions of patients in both the training set (**D**) and the validation set (**G**) are displayed, alongside the expression levels of key genes. **E** and **H** Survival curves for the high-risk and low-risk groups in both the training (**E**) and validation (**H**) sets. **F** and **I** The predictive performance of the risk model on the 1, 3, and 5-year overall survival of HCC patients in high-risk and low-risk groups across both the training set (**F**) and the validation set (**I**)

By intersecting the DEGs between tumorous and non-tumorous liver samples with the ACRRGs(Fig. 2A), we identified 35 overlapping genes (ACRRDEGs) for subsequent analysis. The contribution of the 35 ACRRDEGs to overall survival (OS) in HCC patients was evaluated using the Least Absolute Shrinkage and Selection Operator (LASSO) regression algorithm to mitigate multicollinearity. This approach identified MORF4L1, HDAC1, VPS72, and RUVBL2 as pivotal prognostic indicators (Fig. 2B, C, Table S4).A

risk-scoring model was developed based on the expression levels of these genes, employing the following equation for risk score calculation: Risk Score = (0.26 × Expression Level of MORF4L1) + (0.25 × Expression Level of RUVBL2) + (0.23 × Expression Level of HDAC1) + (0.15 × Expression Level of VPS72). The median risk score was used to categorize patients into high- and low-risk groups, with the former showing a significantly greater mortality(Fig. 2D). Kaplan–Meier analysis confirmed longer OS in the low-risk group (p = 0.0002, Fig. 2E). The model demonstrated substantial predictive accuracy for OS, as.

evidenced by area under the curve (AUC) values of the ROC curves (Fig. 2F). Validation using the GSE14520 dataset from the GEO database corroborated the model's efficacy (Fig. 2G–I).

### Development of a prognostic nomogram for hepatocellular carcinoma using risk score and clinical factors

**Fig. 3:**
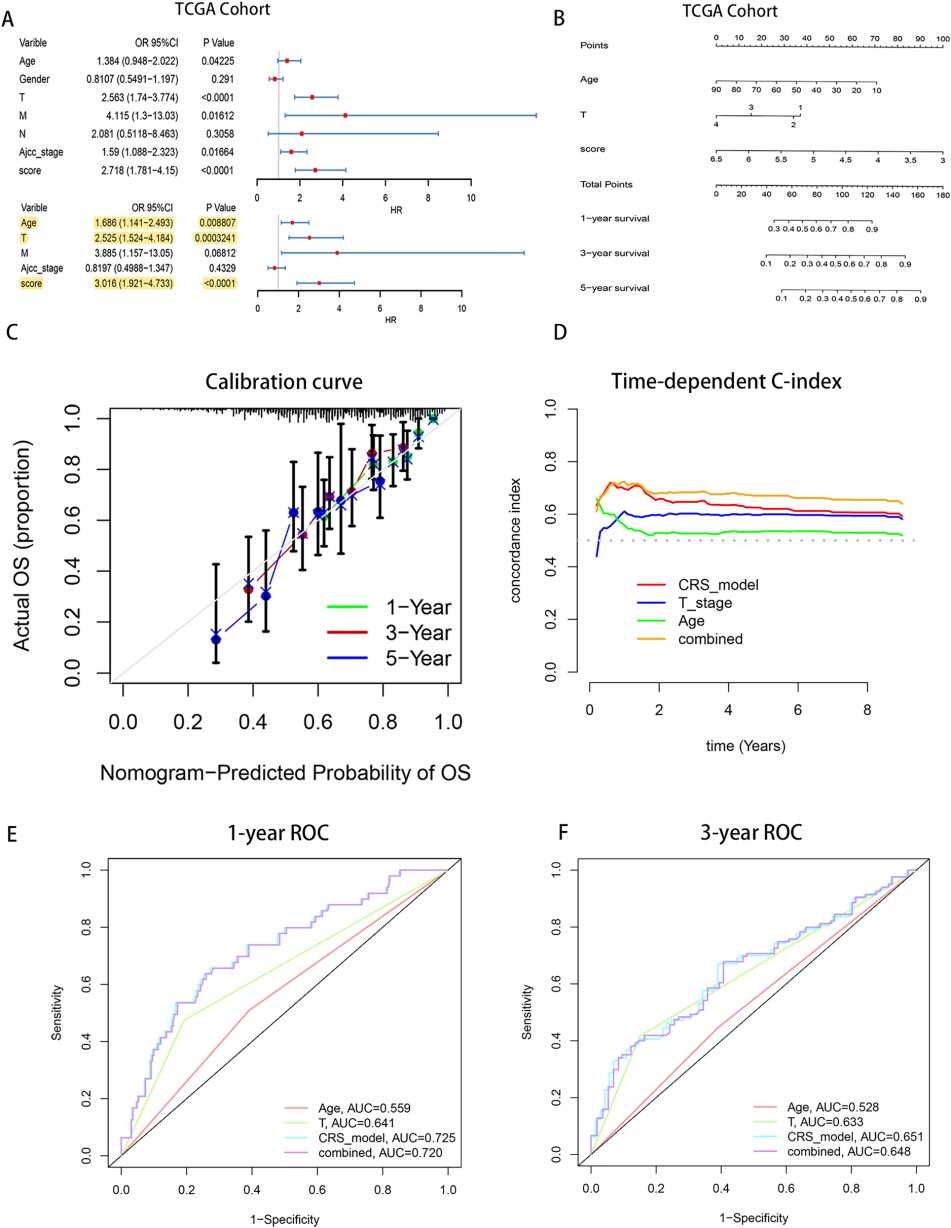
The nomogram, established by integrating risk scores with clinical indicators, demonstrates an improved predictive capability for the prognosis of HCC patients. **A** Cox univariate and multivariate analyses were conducted on clinical factors such as age, gender, tumor TNM staging, and risk score. **B** A nomogram that utilizes age, tumor T stage, and risk score was constructed to predict the 1-, 3-, and 5-year survival rates of HCC patients. (**C**) Calibration curves of the nomogram for predicting the overall survival rates at 1, 3, and 5 years for HCC patients compared to actual survival rates. **D** The concordance index (CI) over time of the risk prediction model and clinical univariate models such as T staging, age, and a model combining these factors. **E** and **F** Receiver operating characteristic (ROC) curves for the T stage model, age model, risk score model, and a comprehensive model combining all three, in predicting the overall survival at 1 and 3 years for HCC patients

Subsequent Cox regression analysis within the TCGA cohort established the risk score, alongside age and T stage, as independent prognostic factors for OS in HCC patients (Fig. 3A). A prognostic nomogram integrating these factors exhibited superior predictive.

performance compared to each individual factor (Fig. 3B). Calibration curves indicated high congruence between the nomogram's predictions and actual survival outcomes, underscoring the model’s predictive reliability (Fig. 3C). Time-dependent C-index and ROC analyses further demonstrated that the nomogram outperforms single clinical predictors in forecasting HCC prognosis (Figs. 3D–F).

### ATP-dependent chromatin remodeling-related genes drive tumor stemness and modulate the immune microenvironment in HCC

**Fig. 4:**
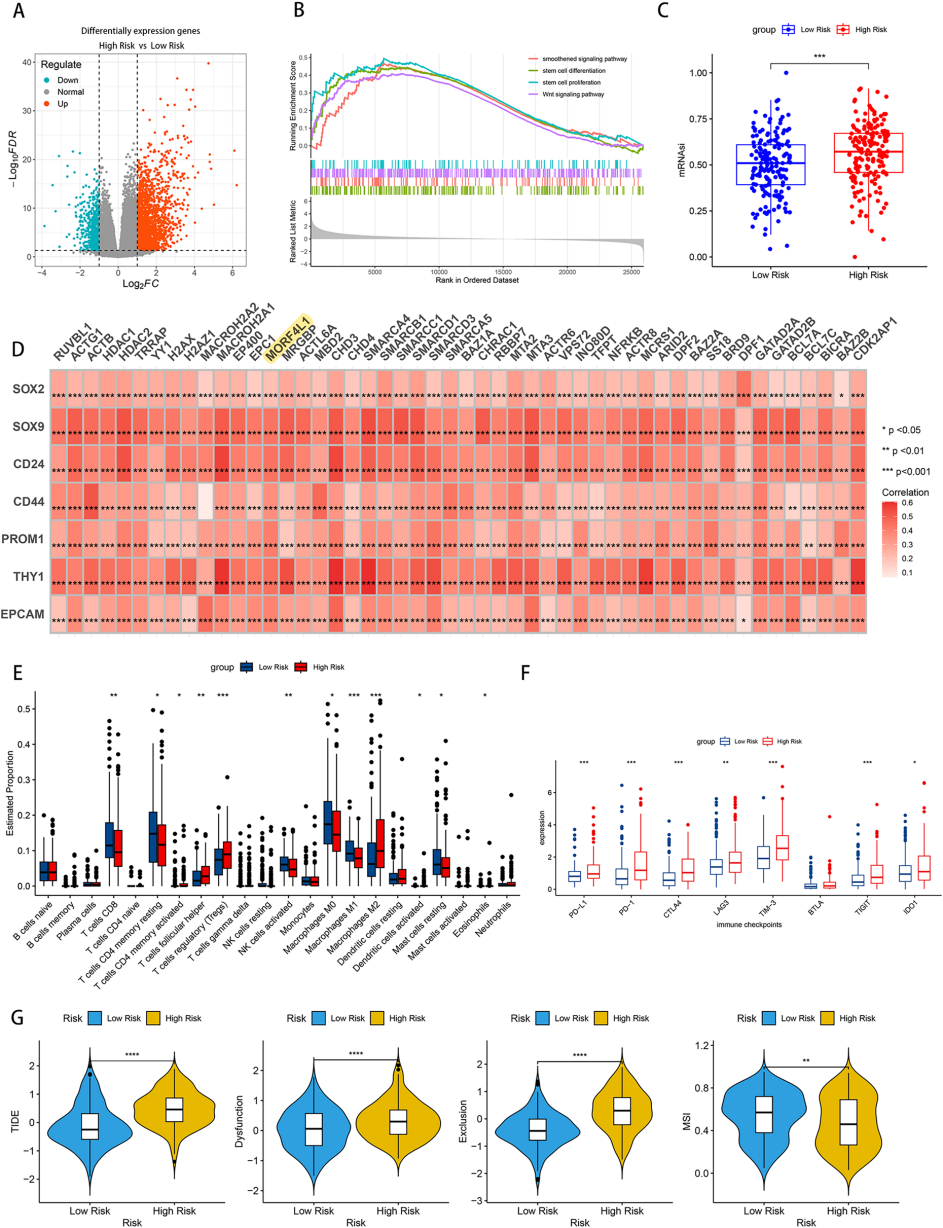
ACRRGs are associated with the stemness and immune escape of HCC. **A** A volcano plot illustrating the differential genes (DEGs) between the high-risk and low-risk groups from TCGA cohort. **B** Results from the Gene Set Enrichment Analysis (GSEA) conducted on DEGs. **C** The distribution of the stemness index (mRNAsi) between the high and low-risk groups. **D** Correlation analysis between ACRRGs and tumor stemness markers. **E** The content of 22 immune cells in the high and low-risk groups was predicted using the CIBERSORT algorithm. **F** The relative expression levels of immune checkpoints in the high and low-risk groups. **G** The response of HCC patients in the high and low-risk groups to immunotherapy was predicted using TIDE

To elucidate the potential mechanisms by which ATP-dependent chromatin remodeling affects HCC prognosis, we initially analyzed datasets from high- and low-risk groups using the DESeq2 R package, aiming to identify differentially expressed genes (Fig. 4A). These genes were subsequently analyzed through GSEA. The results highlighted an upregulation of key pathways such as the 'smoothened signaling pathway,' 'stem cell differentiation,' 'stem cell proliferation,' and 'Wnt signaling pathway' in the high-risk group (Fig. 4B), implying an association between ACRRGs and tumorigenic stemness. To quantify this association, we employed the mRNA Expression-Based Stemness Index (mRNAsi), a novel metric assessing oncogenic dedifferentiation levels [10]. Notably, the high-risk group exhibited a markedly elevated stemness index (Fig. 4C). Further, Pearson correlation analysis underscored a significant positive relationship between ACRRGs’s expression and liver cancer stem cell markers (Fig. 4D), strengthening the evidence that ACRRGs contribute to HCC stemness. To ascertain the influence of ATP-dependent chromatin remodeling on HCC’s immune milieu, we analyzed immune cell compositions and scores in both risk groups using CIBERSORT and the ESTIMATE algorithm. This analysis revealed reduced 'CD8 T cells,'

'Activated NK cells,' and 'M1 Macrophages' presence in the high-risk group, coupled with diminished 'immuneScore' and 'StromalScore' (Fig. 4E and Figure S4). Investigation into immune checkpoint expression showed elevated levels of PD-1, PD-L1, CTLA4, LAG3, TIM3, and IDO1 in the high-risk group (Fig. 4F), suggesting ACRRGs' involvement in immune escape. Utilizing the TIDE algorithm, we predicted a lower likelihood of responsiveness to immunotherapy in the high-risk group, indicated by elevated 'TIDE,' 'Dysfunction,' and 'Exclusion' scores, albeit with a lower MSI score (Fig. 4G). These findings point towards a complex interplay between ACRRGs and the immune microenvironment, potentially influencing HCC's immunotherapeutic response.

### Single-cell analysis reveals differentiation trajectories and the pivotal role of MORF4L1 in hepatocellular carcinoma stem cells

**Fig. 5:**
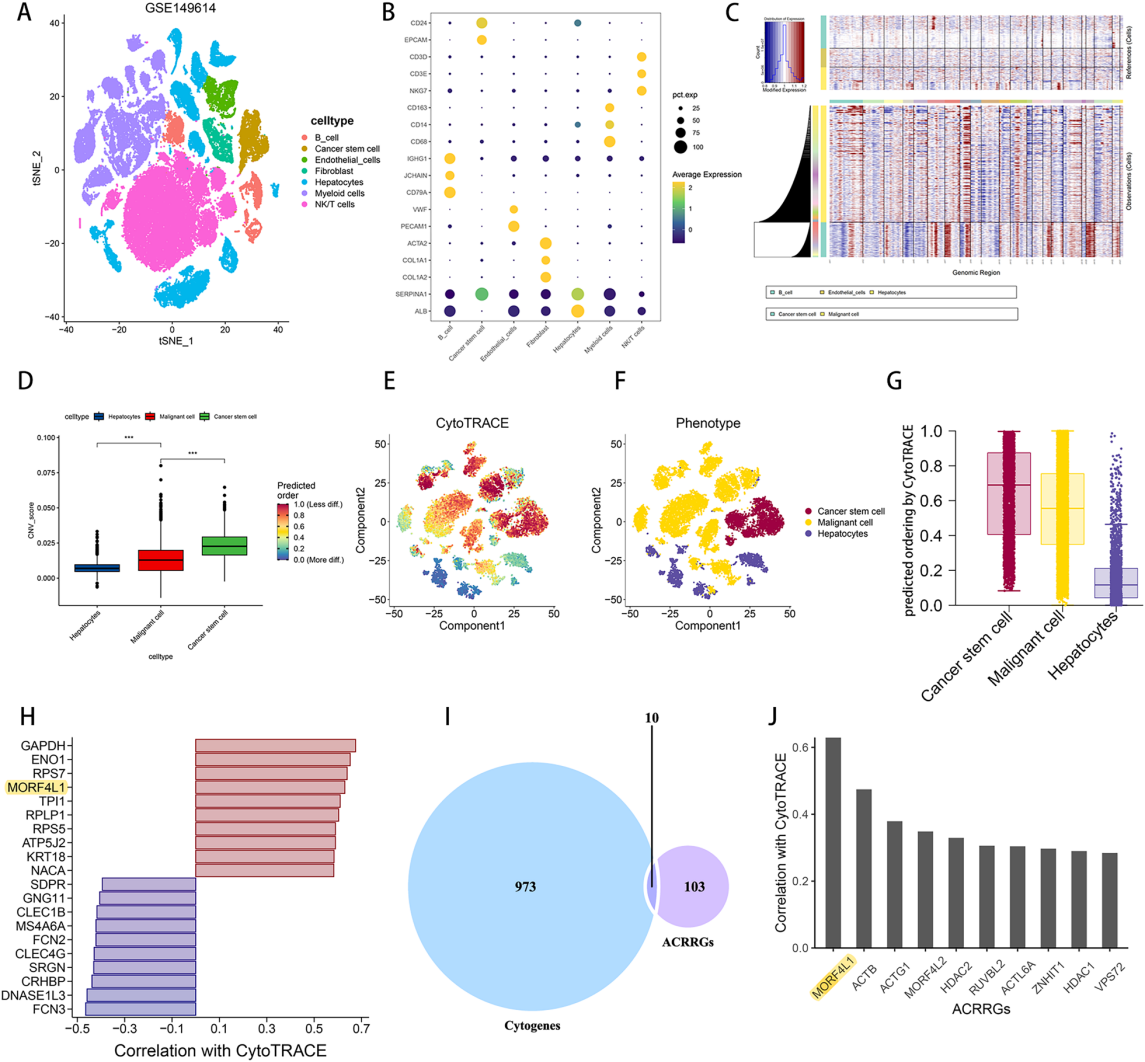
Cancer stem cells have been identified, with MORF4L1 potentially being a key factor in promoting HCC stemness. **A** Identification of cell clusters using single-cell data from GSE149614. **B** Molecular markers corresponding to cell clustering. **C** Copy number variations (CNVs) in cancer stem cells (CSCs), malignant cells, hepatocytes, and reference cell groups (endothelial cells and B cells). **D** CNV scores among hepatocytes, malignant cells, and CSCs. **E**, **F**, and **G** Pseudotime analysis results for hepatocytes, malignant cells, and CSCs. **H** The most significantly changed genes (Cytogenes) during the progression from hepatocytes to CSCs. **I** Intersection of Cytogenes and ACRRGs yields key stemness-regulating genes. **J** Correlation analysis of key genes with the progression of stemness in HCC

Through the Seurat workflow, we processed the single-cell dataset GSE149614, implementing steps such as quality control, data standardization,and normalization, removing the influence of cell cycle factors, dimensionality reduction, clustering, and cell identification. We identified seven major cell types (Figs. 5A, B): Hepatocytes (ALB, SERPINA1), T/NK cells (CD3E, CD3D, NKG7), Myeloid cells (CD68, CD14, CD163), Fibroblasts (ACAT2, COL1A1, COL1A2), Endothelial cells (VWF, PECAM1), CSCs (EPCAM, CD24), and B cells (IGHG1, JCHAIN, CD79A). Following this, we classified Hepatocytes into malignant cells and hepatocytes based on whether their origin was from tumor or normal liver tissue. CSCs are primarily distributed in primary HCC lesions, portal vein tumor thrombi, and metastatic lymph nodes. Additionally, the proportion of CSCs is higher in advanced-stage HCC compared to early-stage HCC(Supplementary File 2). This observation highlights the potential relationship between CSC abundance and HCC progression.Copy number variations (CNVs) are implicated in influencing both the progression and the maintenance of stemness characteristics in a variety of tumors [11–13]. Through CNV.

analysis (Figs. 5C, D), we observed that CSCs exhibited the highest CNV mutations, followed by malignant cells, with the least mutations found in hepatocytes. This suggests that CNV mutations could impact the progression of HCC stemness. Through the CytoTRACE analysis, we compared the differentiation potential of hepatocytes, malignant cells, and cancer stem cells (CSCs). We found that CSCs exhibited the highest differentiation potential, followed by malignant cells, and hepatocytes showed the lowest(Fig. 5E–G), which further validates the credibility of our identification of cancer stem cells. In the Cytotrace analysis, the score of each cytogene measures its correlation with cell differentiation and stemness. Among these cytogenes, we identified some genes previously characterized as ACRRGs in this article that are strongly associated with HCC stemness, with MORF4L1 being the most notable. (5H-J).

### MORF4L1 is upregulated in HCC and enhances the stem-like characteristics of HCC cells in vitro

**Fig. 6:**
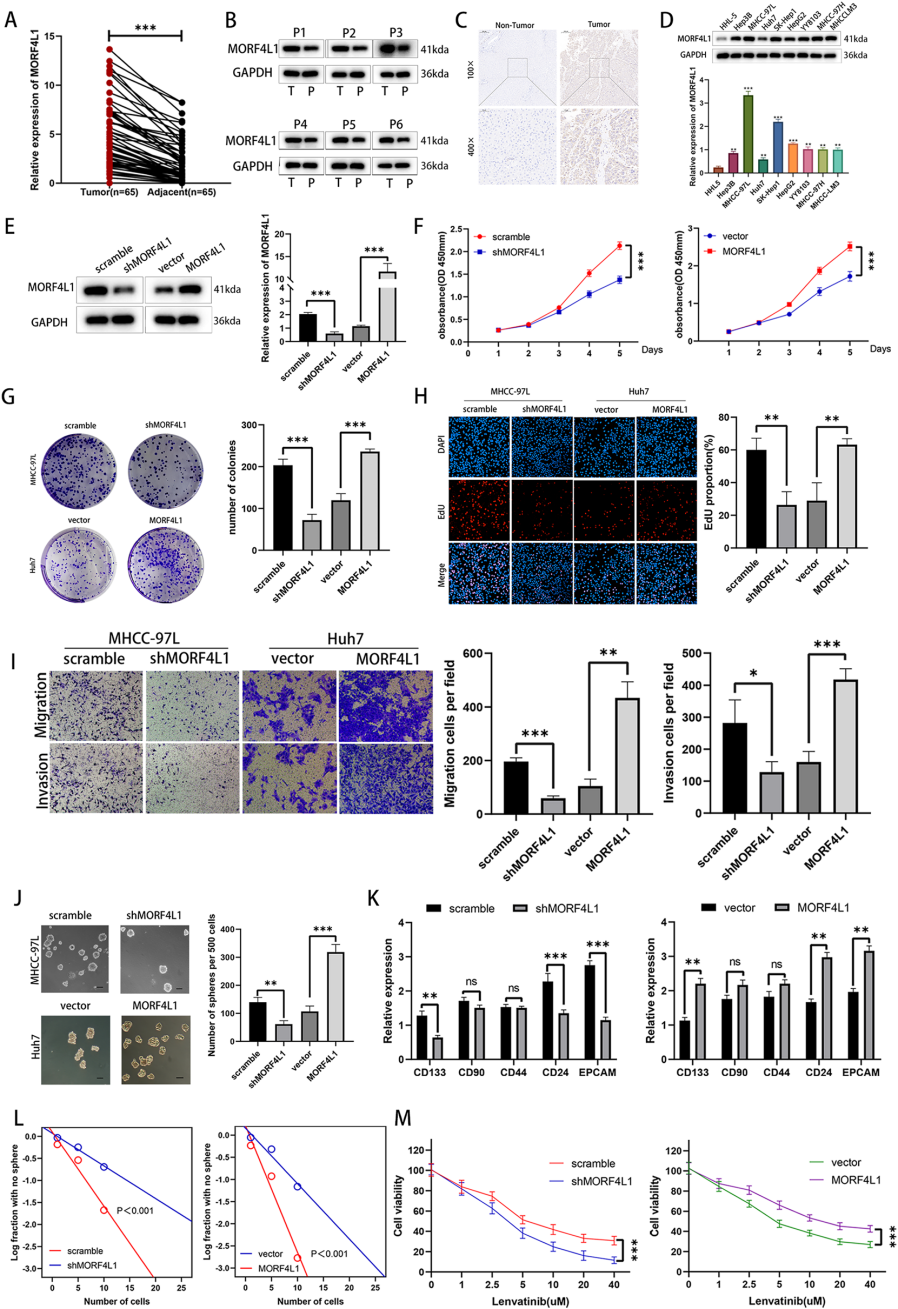
MORF4L1 is expressed at higher levels in HCC and promotes stem-like characteristics of HCC cell lines in vitro. **A** PCR was employed to assess the relative expression of MORF4L1 mRNA in 65 pairs of HCC and adjacent non-tumor liver tissues. **B** and **C** Western blot and immunohistochemistry were used to measure the protein expression levels of MORF4L1 in HCC and adjacent non-tumor liver tissues. **D** The relative mRNA and protein expression levels of MORF4L1 were determined in eight HCC cell lines and one normal liver cell line(HHL5). **E** Western blot and qRT-PCR were used to verify the efficiency of MORF4L1 knockdown and overexpression. **F**–**H** Plate cloning, CCK8, and EdU assays were utilized to assess cell proliferation capabilities. **I** Transwell assay was used to evaluate cell migration and invasion abilities. **J** Representative images of spheroids formed by HCC cells with MORF4L1 knockdown, MORF4L1 overexpression, and their control cells. Scale bar: 50 μm. **K** mRNA expression levels of cancer stem cells (CSCs) markers changed in the indicated cells. **L** Results from limiting dilution assays in the indicated cells. **M** Cell viability assays were conducted on the indicated cells with varying concentrations of lenvatinib. Untreated cells served as the baseline with 100% viability

Through Real-Time Quantitative Reverse Transcription PCR (qRT-PCR), Western Blot, and immunohistochemistry analyses on tumor and adjacent normal liver tissues, we observed significantly higher MORF4L1 expression levels in HCC tissues at both mRNA and protein levels (Figs. 6A–C). This upregulation was confirmed across eight HCC cell lines compared to a normal liver cell line, with MHCC-97L and Huh7 cells displaying the highest and lowest MORF4L1 levels, respectively (Fig. 6D). Lentiviral transfection experiments to modulate MORF4L1 expression in these cell lines demonstrated that MORF4L1 downregulation inhibited, while its upregulation promoted, cellular proliferation, migration, and invasion, as evidenced by plate cloning, CCK8, EdU, and Transwell assays (Fig. 6E–I).

Sphere formation assays indicated that MORF4L1 silencing reduced, whereas its overexpression increased, the number of spheres formed, suggesting MORF4L1's involvement in maintaining HCC stemness (Fig. 6J). This was further supported by the significant changes in cancer stem cell (CSC) marker expression (CD133, CD24, EPCAM) corresponding to MORF4L1 expression levels (Fig. 6K). Limiting dilution assays revealed that MORF4L1 manipulation significantly altered sphere formation efficiency, aligning with its role in stem cell self-renewal (Fig. 6L). Moreover,CSCs have been reported to be involved in resistance to lenvatinib [14, 15], and response assays to lenvatinib demonstrated that MORF4L1 modulation significantly impacted drug sensitivity in HCC cell lines (Fig. 6M).

### MORF4L1 promotes tumorigenesis, metastasis, and recurrence in HCC In Vivo

**Fig. 7:**
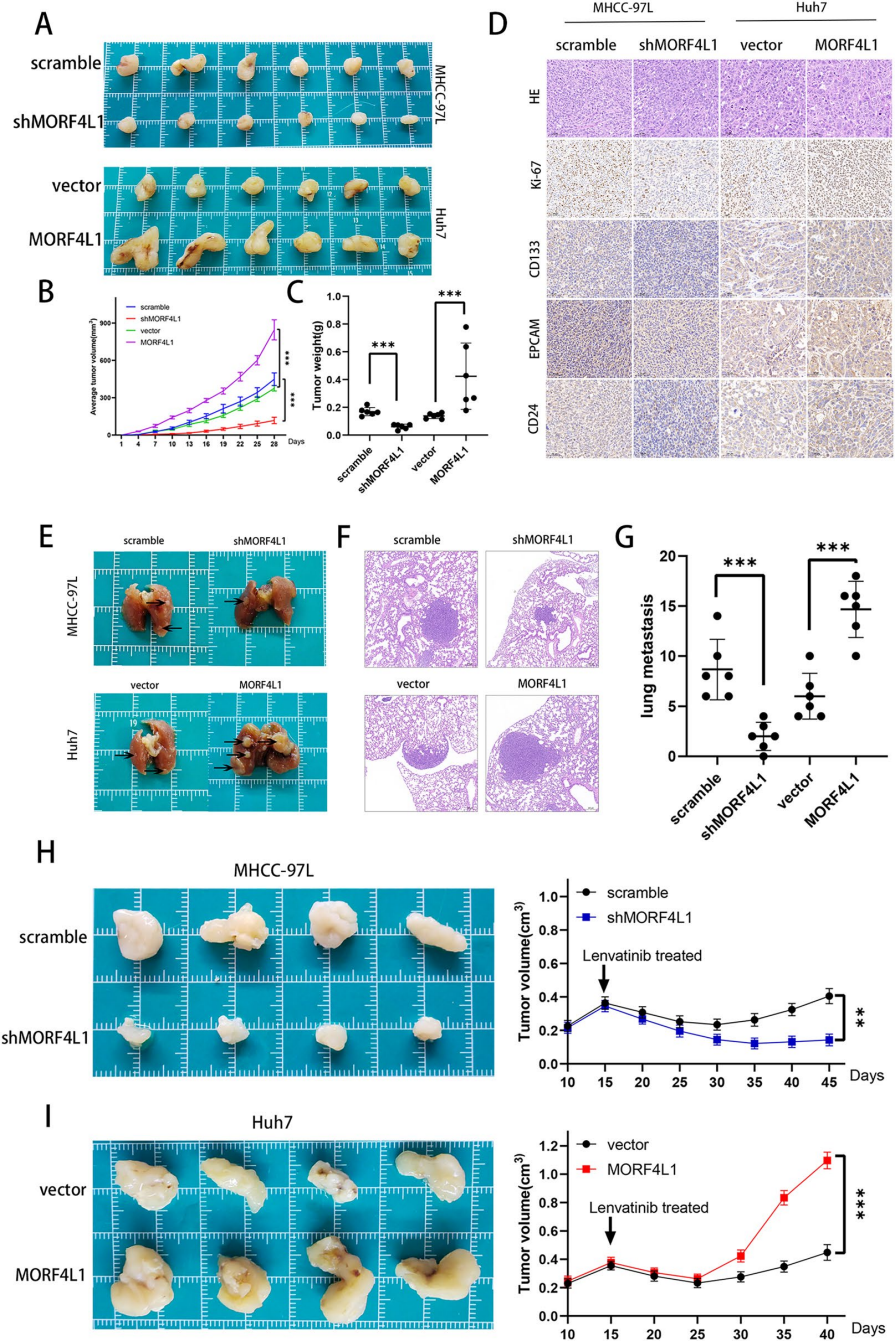
MORF4L1 promotes tumorigenesis, metastasis and recurrence in HCC in vivo. **A** Images show subcutaneous xenograft tumors in nude mice formed by injecting the indicated cells. **B** Volume growth curves of subcutaneous xenograft tumors. **C** Weights of the subcutaneous xenograft tumors. **D** Histological (H&E staining) and immunohistochemical staining for KI67, CD133, EPCAM and CD24 were performed on subcutaneous xenograft tumors. **E** and **F** Representative images of lung metastases from tail vein injection of indicated cells, along with H&E staining photographs of these metastases. **G** Counts of metastatic foci in the lungs. **H** and **I** Subcutaneous injection of MORF4L1-knockdown or MORF4L1-overexpressing cells into nude mice. Upon tumors reaching an average volume of 0.35 cm^3^ (day 15), mice received daily oral administration of lenvatinib (20 mg/kg) for a week. On the left are representative images of subcutaneous tumors, with corresponding tumor growth curves on the right

In a subcutaneous tumor model using nude mice, MORF4L1 overexpression accelerated tumor growth and tumorigenesis, whereas its silencing had an inhibitory effect (Fig. 7A–C). Immunohistochemistry revealed altered expression levels of KI67 and stemness markers (CD133, EPCAM, CD24) corresponding to MORF4L1 levels(Fig. 7D). A lung metastasis model further confirmed MORF4L1's role in enhancing HCC metastasis (Fig. 7E–G). Lenvatinib treatment studies demonstrated MORF4L1's influence on drug resistance and tumor recurrence, with knockdown cells showing sustained tumor growth inhibition (Fig. 7H–I).

### MORF4L1 enhances hepatocellular carcinoma stemness by activating the hedgehog signaling pathway

**Fig. 8:**
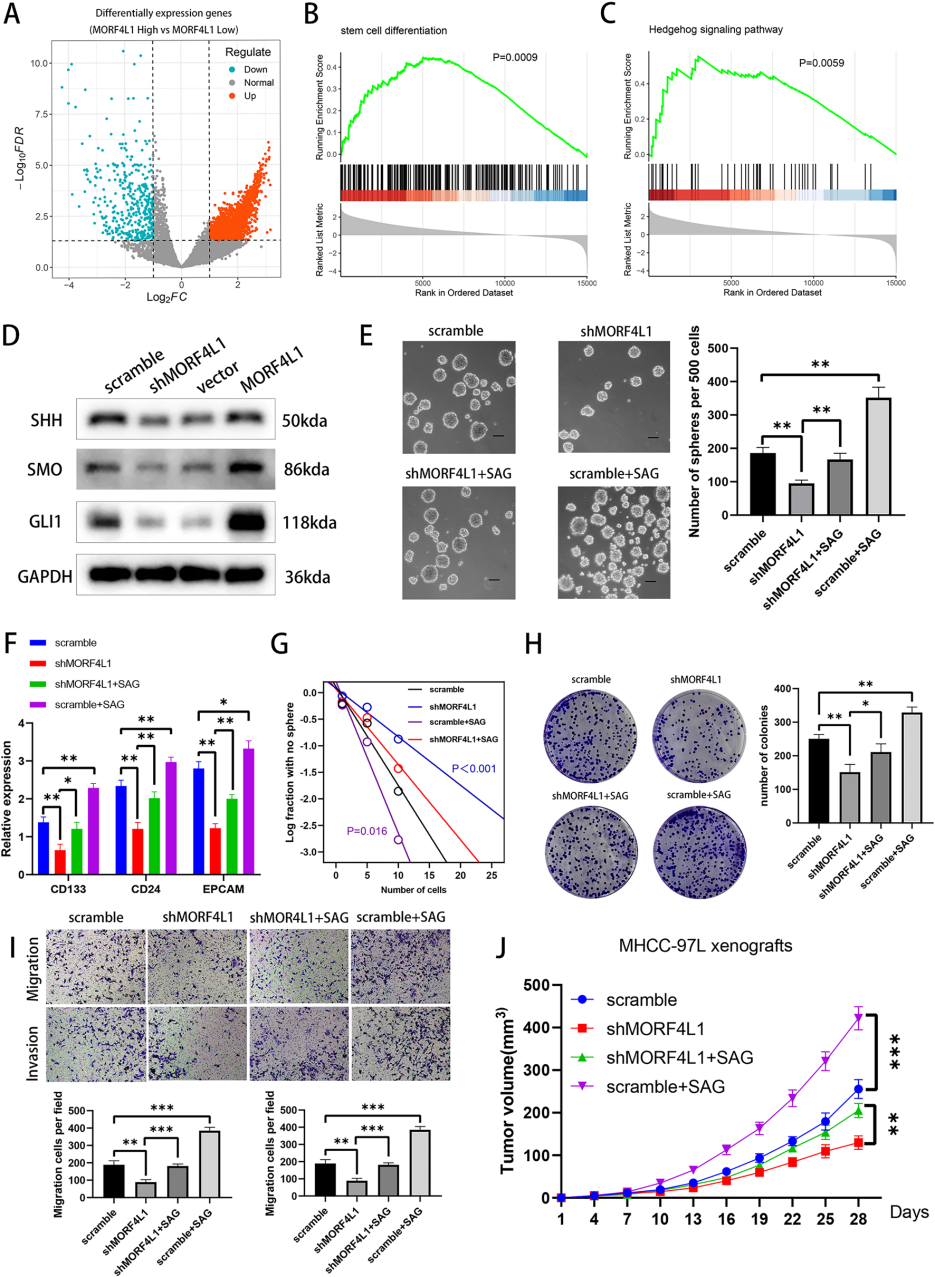
MORF4L1 activates the Hedgehog pathway to promote stemness in HCC. **A** Volcano plot depicting differential gene expression (DEGs) between groups with high and low MORF4L1 expression in TCGA-LIHC. **B** and **C** Pathways identified from GSEA analysis of DEGs. **D** Western blot analysis for critical Hedgehog pathway proteins in HCC cells with MORF4L1 knockdown or overexpression. **E** Representative images of spheroids by indicated cells. Scale bar: 50 μm. **F** RT-PCR analysis for relative mRNA expression levels of CSC markers in indicated cells. **G** Limiting dilution assay reveals sphere formation frequency in indicated cells. **H** and **I** Assessments of plate colony formation and transwell migration/invasion assays in indicated cells. **J** Growth curves of subcutaneous xenograft tumors in nude mice injected with indicated HCC cells. Data are presented as means ± SD. (*p < 0.05; **p < 0.01; *p < 0.001)

Differential gene expression analysis of TCGA HCC samples stratified by MORF4L1 expression highlighted enriched 'stem cell differentiation' and 'Hedgehog signaling pathway' genes (Fig. 8A–C). The Hedgehog pathway is known to be associated with the proliferation and differentiation of CSCs [16, 17]. Western Blot analysis confirmed that MORF4L1 levels directly affected the expression of key Hedgehog pathway proteins (SHH, SMO, GLI1) (Fig. 8D). Interventional experiments using the SHH pathway activator (SAG) on MORF4L1-knockdown cells restored spheroid formation capacity and in vivo tumorigenicity, establishing MORF4L1’s pivotal role in HCC stemness through the Hedgehog pathway activation (Figs. 8E–J).

## Discussion

The high mortality rate of HCC is primarily attributed to late-stage diagnosis [3], emphasizing the importance of early diagnosis and prognosis prediction. Biomarkers like AFP and PIVKA-II have improved early diagnosis rates of HCC due to their high sensitivity and specificity. However, a significant proportion of HCC patients remain negative for these markers [18–21], underscoring the need for additional indicators to enhance early detection models. As a result, predictive modeling using machine learning and bioinformatics has become increasingly popular. These models include imaging-based approaches (e.g., CT, MRI, ultrasound) [22–25], gene-focused models involving ferroptosis- and cuproptosis-related genes [26–29], and immune microenvironment models centering on tumor-associated macrophages (TAMs) and cancer-associated fibroblasts (CAFs) [30–33].

ATP-dependent chromatin remodeling has emerged as a novel epigenetic mechanism that plays a pivotal role in various diseases and cancers [34]. Recent studies, such as Muran Xiao's research, have demonstrated that chromatin remodeling components like BRD9 influence stem cell differentiation and tumor progression [35]. Alterations in the SWI/SNF complex have also been linked to rare neural tumors, such as H3.3K27M diffuse intrinsic pontine gliomas and clear cell meningiomas, with targeting these components showing potential for suppressing tumor growth [36, 37]. Furthermore, ATP-dependent chromatin remodeling significantly impacts tumor progression in cancers such as prostate, breast, pancreatic, and lung [6, 38–41]. However, its role in HCC remains unexplored.

In this study, we examined the expression of ATP-dependent chromatin remodeling-related genes (ACRRGs) in HCC and their prognostic significance. Analysis of public databases revealed that most ACRRGs are highly expressed in HCC tissues and correlate with overall survival. Using machine learning, core genes (MORF4L1, HDAC1, RUVBL2, VPS72) were identified to construct a risk prediction model and nomogram, demonstrating high predictive accuracy in both training and validation cohorts. This work introduces a novel, reliable prognostic model for HCC based on ACRRGs.

Cancer stem cells (CSCs), a minor subset within tumors, are characterized by robust self-renewal and tumorigenic potential. They contribute to tumor initiation, metastasis, drug resistance, and immune evasion [42–44], making them promising therapeutic targets [45–47]. Our study highlights the association between ACRRGs and CSC-related markers (e.g., CD133, CD24, EPCAM) and the stemness index mRNA (mRNAsi). HCC samples with high ACRRG scores exhibited reduced levels of CD8 + T cells, NK cells, and M1 macrophages but increased levels of Treg cells and M2 macrophages, indicating a role for ACRRGs in modulating the HCC immune microenvironment.

Single-cell RNA sequencing (scRNA-seq) has provided new tools for reconstructing differentiation trajectories. However, traditional methods like Monocle face challenges in identifying cells exhibiting the highest stemness [48]. CytoTRACE, a more advanced tool, assesses differentiation states using gene counts, predicting differentiation potential without requiring continuous developmental processes [49]. In our study, CytoTRACE analysis revealed a differentiation potential hierarchy among liver cells: CSCs exhibited the highest differentiation potential, followed by malignant liver cells, and then normal hepatocytes. This finding suggests that the carcinogenic process in HCC is associated with enhanced differentiation potential and stem-like characteristics. Cross-referencing CytoTRACE results with ACRRGs identified MORF4L1 as a critical gene influencing stem-like features in HCC.

MORF4L1, located at chromosome 15q25.2, is involved in epigenetic regulation and chromatin remodeling, influencing DNA repair, cell cycle regulation, and tumorigenesis. It has also been implicated in mitochondrial function and metabolic regulation in liver diseases, including HCC precursors like non-alcoholic steatohepatitis [50]. Additionally, MORF4L1 interacts with proteins such as ASH1L, activating histone methyltransferase activity and potentially altering tumor chromatin structure [51]. Our study demonstrates that MORF4L1 promotes HCC initiation and progression through in vitro sphere formation and in vivo tumor models. We also found that MORF4L1 enhances drug resistance and metastasis in HCC, confirmed through lenvatinib treatment and lung metastasis models. Downstream pathway analysis revealed that MORF4L1 activates the Hedgehog signaling pathway, enhancing stem-like properties in HCC. These findings establish MORF4L1 as a potential therapeutic target in HCC.

In conclusion, our research highlights the prognostic significance of ATP-dependent chromatin remodeling-related genes in HCC. We developed an effective prognostic model and demonstrated its utility in predicting immunotherapy response. Furthermore, MORF4L1 was identified as a key promoter of stem-like properties in HCC through the Hedgehog pathway, presenting a promising new therapeutic target.

## Supplementary Information

Supplementary material 1

## Data Availability

No datasets were generated or analysed during the current study.
